# Design of DNA Pooling to Allow Incorporation of Covariates in Rare Variants Analysis

**DOI:** 10.1371/journal.pone.0114523

**Published:** 2014-12-08

**Authors:** Weihua Guan, Chun Li

**Affiliations:** 1 Division of Biostatistics, School of Public Health, University of Minnesota, Minneapolis, MN, United States of America; 2 Department of Epidemiology and Biostatistics, Case Western Reserve University, Cleveland, OH, United States of America; The University of Hong Kong, Hong Kong

## Abstract

**Background:**

Rapid advances in next-generation sequencing technologies facilitate genetic association studies of an increasingly wide array of rare variants. To capture the rare or less common variants, a large number of individuals will be needed. However, the cost of a large scale study using whole genome or exome sequencing is still high. DNA pooling can serve as a cost-effective approach, but with a potential limitation that the identity of individual genomes would be lost and therefore individual characteristics and environmental factors could not be adjusted in association analysis, which may result in power loss and a biased estimate of genetic effect.

**Methods:**

For case-control studies, we propose a design strategy for pool creation and an analysis strategy that allows covariate adjustment, using multiple imputation technique.

**Results:**

Simulations show that our approach can obtain reasonable estimate for genotypic effect with only slight loss of power compared to the much more expensive approach of sequencing individual genomes.

**Conclusion:**

Our design and analysis strategies enable more powerful and cost-effective sequencing studies of complex diseases, while allowing incorporation of covariate adjustment.

## Introduction

With the recent advances in next-generation sequencing (NGS) technology, it has become feasible to explore the rare and less common variants in individual genomes with high throughput screening, for example, the 1000 Genomes Project (http://www.1000genomes.org/, [1]), the UK10K project (www.uk10k.org), and the NHLBI GO Exome Sequencing Project (ESP) (https://esp.gs.washington.edu/, [2]). These projects allow investigators to conduct a survey of both common and rare variants in well phenotyped populations, and increase the chance of discovery for disease-causing variants. However, the cost of whole genome and whole exome sequencing is still high. To be able to identify rare or less common variants, a large number of samples need to be sequenced. In addition, the throughout of the latest sequencer is very high that several billions of reads can be generated from a single flow cell. For a sequencing study of a small targeted region, it translates to many thousand-fold coverage if each individual is sequenced per lane, which is far greater than needed to obtain accurate calls for the genotypes. Therefore, cost-effective methods and study designs will be helpful to increase the size of sequencing studies and power of the association tests while fully using the capacity of the sequencer. One choice of such designs is DNA pooling [3]–[6], which pools a number of individual DNAs to sequence as a single sample.

DNA pooling can efficiently use sequencing depth while reducing the cost of target capture and library preparation, especially in targeted re-sequencing studies for regions of tens to hundreds of kilobases. In addition, sequencing pooled DNA samples can provide better SNP discovery and more accurate allele frequency estimate than individual sequencing, even with presence of sequencing errors and unequal contribution of individuals to the pool. [7]–[10] Comparing to the intensity measure in microarray experiments, the read counts from sequencing can be modeled using binomial distribution and allow better inference on individual-level genotypes from pooled DNA samples.

Pooling can be done with tagging, which multiplexes samples with barcodes prior to pooling [11], [12], and allows identification of individual samples in the pool. However, indexing individual DNA samples will add to the labor and cost for processing the extra barcode sequence. Sequencing errors can also lead to non-perfect match in the index sequence which can reduce the total number of reads, or quality of data if mismatches are allowed. In this paper, we will consider DNA pooling of non-barcoded DNA samples, and develop novel statistical method for pool creation and analysis of pooled sequence data.

Weinberg and Umbach [13] showed that in a case-control study, well-modeled statistical tests for pooled samples lose very little statistical power compared to the individual-based analysis. Statistical methods have also been developed specifically for sequencing study of pooled samples ([14], [15]). However, a potential limitation of the pooling strategy is that the identity of individual genomes would be lost and therefore individual characteristics and environmental factors cannot be adjusted in association tests. Such limitation may result in power loss, and even false positives in presence of confounding effect (e.g., ethnicity). Weinberg and Umbach [13] suggested exact match on covariates when pooling samples, but such matching criteria is often difficult to achieve, especially when the number of covariates is large and the variables are not discrete.

In this paper, we propose a design strategy for pool creation in case-control sequencing studies, which does not require exact match of covariate values, and use multiple imputation technique to impute and analyze individual-level genotype and covariates for SNP-disease association. We will use computer simulations to validate our approach, and compare its statistical power to that of individual-based analysis and pool-based analysis without covariate adjustment. We hope this new design and analysis strategy can provide an alternative approach to allow more powerful and cost-effective sequencing studies of complex diseases.

## Methods

For case-control sequencing studies, we propose a design and analysis strategy for DNA pooling that can greatly reduce the cost of sequencing and also allow covariate adjustment for SNP-disease association. Our method includes three steps:

1. *Pool creation*: Case and control samples will be grouped according to the similarity of their characteristics (e.g., age, sex, ethnicity or principal components, etc.). Samples with similar characteristics will be pooled for sequencing.
2. *Genotype Imputation*: Assuming a random binomial process, we calculate the probabilities of genotype for each sample in the pool, given the total number of reads from sequencing. The genotypes will then be randomly drawn following these probabilities.
3. *Association test*: We use logistic regression to assess the association between candidate markers and disease status, controlling for other genetic or environmental factors. Using multiple imputation technique [16], we repeat 2) and 3) multiple times to obtain valid estimates for the genetic effects.

### Sample matching and pool creation

We will pool samples by their similarity in covariates. When the covariates are all categorical and have a limited number of categories, an exact match can be applied. In this paper, we consider a matching approach based on the predicted probability of being affected.

Let *y* denote the observed disease status (1 = affected, 0 = unaffected), *g* be the genetic marker, and *Z*  =  (*z*_1_, …, *z_k_*) be the vector of covariates. We fit a logistic regression model of the disease risk on the covariates (without the genetic marker)
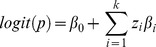
 and calculate the predicted probability for sample *s* as
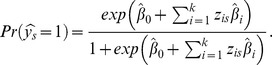



We then create the pools by dividing samples into multiple groups according to quantiles of the predicted probabilities. (see Appendix for details)

### Genotype Imputation

DNA pools will be created with approximately equal amount from each sample. Sequencing will be carried out on the pooled DNA samples. Because sequencing error in next-generation sequencing can be high even after quality control filtering and may confound the disease-marker association, we incorporate the “blocked pooling” design suggested by Wang et al. [9]. Using this approach, each pooled sample is barcoded and multiple indexed DNA pools will be sequenced in one lane. This blocked design allows accurate estimation of both locus-specific sequencing error rate and allele frequency.

We consider a DNA pool with *K* individuals. Let *p* and *q* = 1 - *p* be the frequencies of alleles *a* and *A*, respectively. Let 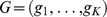 be the genotype for the *K* subjects in the pool, and 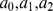 be the number of genotypes *AA*, *Aa*, *aa* in *G*, respectively. Let 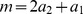 be the number of allele *a* in the pool of 2*K* alleles. Assuming Hardy-Weinberg equilibrium (HWE), *m* follows a binomial distribution Bin(2*K*, *p*). When the variant is less common, *p* is small and 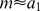. Let 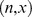 be the observed sequence data for the pool, where *n* is the total number of reads at the locus and *x* the number of reads with allele *a*. Denote *e* the sequencing error rate, and then 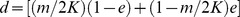 the probability of observing a read of allele *a*. The joint probability:
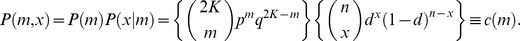
and the posterior distribution of *m* given observed reads *x* is 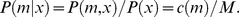
where 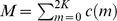. Note that *c*(0) = *c*(2*K*) = 0.

Utilizing the blocked design, the allele frequency *p* and sequence error rate *e* can be jointly estimated using the EM algorithm developed by Wang et al. [9]. When the allele is causal, the HWE can be violated in the entire case-control cohort. The allele frequency is therefore estimated separately for the pools of case samples and pools of control samples, and can be further stratified by the covariate values. (see Appendix)

To impute the genotype for each individual from the pooled sequence data, we draw (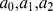)_impute_ as follows:

1. Randomly generate *m* from the distribution 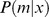, based on the estimated allele frequency *p* and sequence error rate *e*.
2. Given the value of *m*, draw *a*_1(impute)_ from 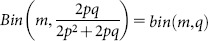, and if *m* – *a*_1(impute)_ is odd, redraw *a*_1(impute)_. Once we have *a*_1_, we calculate *a*_2(impute)_ = (*m* – *a*_1(impute)_)/2 and *a*_0(impute)_ = *K* – *a*_1(impute)_– *a*_2(impute)_.
3. Randomly assign the genotype *AA*, *Aa*, or *aa* to the *K* samples in the pool such that there are *a*_0(impute)_ samples with *AA*, *a*_1(impute)_ samples with *Aa*, and *a*_2(impute)_ samples with *aa*.
4. Repeat (1)-(3) multiple times.

### Association test

After the individual genotypes are imputed from pooled sequence data, standard logistic regression can be used to test for disease-marker association, with the covariates being adjusted in the model. To take into account the uncertainty of imputed data and obtain valid estimates, multiple imputation technique will be applied. Specifically, we will repeat the genotype imputation and association test multiple times (typically 5–10), and combine the results to produce estimated effects and confidence intervals [16].

### Simulations

We simulate case-control data possibly influenced by genotypes (*G*) at a disease locus. We assumed the allele frequency of causal allele at 1%, and an odds ratio (OR) of 1.0 (no association) and 3.5 (disease-causing) as the primary simulation model, but also considered models with different allele frequencies and effect sizes. We randomly sample equal numbers of cases and controls (n = 1000 cases/controls), and consider a pool size of 12. For sequencing of pooled samples, we assume an average coverage of 200X at the disease locus. The actual coverage for each sample is randomly drawn from a normal distribution with standard deviation of 20X. We assume 5 additional risk factors (*Z*) that are associated with the disease with equal or unequal ORs (Table 1).

**Table 1:** Characteristics of simulation models.

Model	n	RAF	corr	OR_g_	OR_z_	α_max_	note
1	1000	.01	0	1.0	(1.5, 1.5, 1.5, 1.5, 1.5)	0	Base model
2	1000	.01	**.1**	1.0	(1.5, 1.5, 1.5, 1.5, 1.5)	0	Correlated G and Z
3	1000	.01	**.1**	1.0	**(1.1, 1.3, 1.9, 2.0, 2.4)**	**100%**	Unequal %sample
4	**5000** $	.01	**.1**	1.0	**(1.1, 1.3, 1.9, 2.0, 2.4)**	0	Large sample size
5	1000	.01	0	3.5	(1.5, 1.5, 1.5, 1.5, 1.5)	0	Base model
6	1000	.01	**.1**	3.5	(1.5, 1.5, 1.5, 1.5, 1.5)	0	Correlated G and Z
7	1000	.01	**.1**	3.5	**([1.5, 1.5, 1.5,]** 1.5, 1.5)*	0	Not all Z observed
8	1000	.01	**.1**	3.5	**(1.1, 1.3, 1.9, 2.0, 2.4)**	0	Varying effect of Z
9	**5000** $	.01	**.1**	3.5	**(1.1, 1.3, 1.9, 2.0, 2.4)**	0	Large sample size
10	1000	.01	**.1**	3.5	**(1.1, 1.3, 1.9, 2.0, 2.4)**	**100%**	Unequal %sample

*. The simulation model consists of 5 covariates, each with OR of 1.5. In analysis, we assume that only the last two covariates are considered.

$. In analysis, a more stringent threshold (10^−4^) is used for significance, compared to other simulation models (.05).

n: number of cases, assuming case:control ratio of 1∶1; RAF: risk allele frequency; OR_g_: odds ratio for risk allele; OR_z_: odds ratios for covariates; corr: correlation coefficient between causal variant and the last covariate; α_max_: variation in sample proportions (see “Methods”). Model 1–4 were simulated under the null hypothesis of no association; and model 5–12 were under the alternative hypothesis. Model 1 and 5 were treated as baseline models, and changes of parameters in other models were highlighted.

In the simulations, we first assume that the genotype is not associated with the disease (model 1–4) and evaluate the type I error of our proposed method; model 5–12 assume an OR of 3.5 for the causal allele to compare power of different methods. We vary the correlation between covariate *Z* and genotype *G*. In model 1 and 5, *Z* are independent risk factors so that the type 1 error of standard pooling method can be correctly controlled without adjusting for any covariates. In the other simulation models, we assume that one of the risk factors (for example, sample ethnicity or a biomarker) is correlated with *G* and serve as a confounder. We also vary the allele frequency and effect size of G and effect size of Z, using simulation settings similar to those in model 5.

We further consider simulation models to reflect possible scenarios of a real study. In practice, we often do not have information on all risk factors. In model 7, we assume that only two of the five risk factors are included in analysis. In model 8, we assume different effect sizes for the risk factors. We also consider a more stringent significance level of 10^−4^ using a large sample size (n = 5000, model 4 and 9) to evaluate the type 1 error and power of proposed method. In model 4 and 10, we simulate situations where the DNA samples are not equally represented in the pool, reflecting potential technical errors in pool creation (see below). At last, we simulate the sequence data with different sequencing errors (.5%, 1%, and 2%), and evaluate the type 1 errors, using model 2 and model 6 as an example.

For each model, we simulate 1,000 datasets (1,000,000 datasets for model 4), and use 5 imputations in our proposed method.

We compare the power of our method (“pool_MI_”) to individual sequencing of all cases and controls (“seq_all_”) and standard DNA pooling without considering other risk factors (“pool_univariate_”), where “seq_all_” would provide the maximum power when budget is not a factor for experiment design. We calculate type 1 error and power as the proportion of simulated replicates where the association p-value is <.05 or 10^−4^ at the locus.

Although equal concentration for individual samples in the pool is desired, in practice their contributions can vary due to technical variability. We further carry out simulations in which the samples have unequal proportions in the pool (model 4 and 10). We randomly generate weight of an individual sample as 1/K*(1+α), where K is the pool size and (1+α) is the scale parameter with α being randomly drawn from (-α_max_, α_max_). In the simulations, we set α_max_ to be 1. The weights are further normalized and used as sampling proportions in the pool. We perform association tests using our approach under the equal proportion assumption, and evaluate type 1 error and power.

## Results

### Type 1 error and power

We first evaluate the false positive rates of our proposed approach and other alternatives (Table 2). When there is no confounding effects (model 1), all methods give false positive rates close to the nominal value of.05. However, when there is a confounder in the model (model 2–4), for example, the variant is an ancestry informative marker and ethnicity is correlated with disease outcome, the standard pooling approach without adjusting for covariates can lead to inflated type 1 error, while the proposed method still maintained the type 1 error at the correct level.

**Table 2:** Type 1 error for multiple-imputation based pooling method (“pool_MI_”), individual sequencing of all samples (“seq_all_”) and pooling without considering other risk factors (“pool_univariate_”).

Model	seq_all_	pool_univariate_	pool_MI_
1	.044	.046	.043
2	.046	.458	.048
3	.058	.368	.060
4	1.1E-4	.040	1.2E-4

The significance level  = .05 for model 1–3, and 10^−4^ for model 4. Number of simulations is 1000 for model 1–3, and 100,000 for model 4.

**Figure 1:**
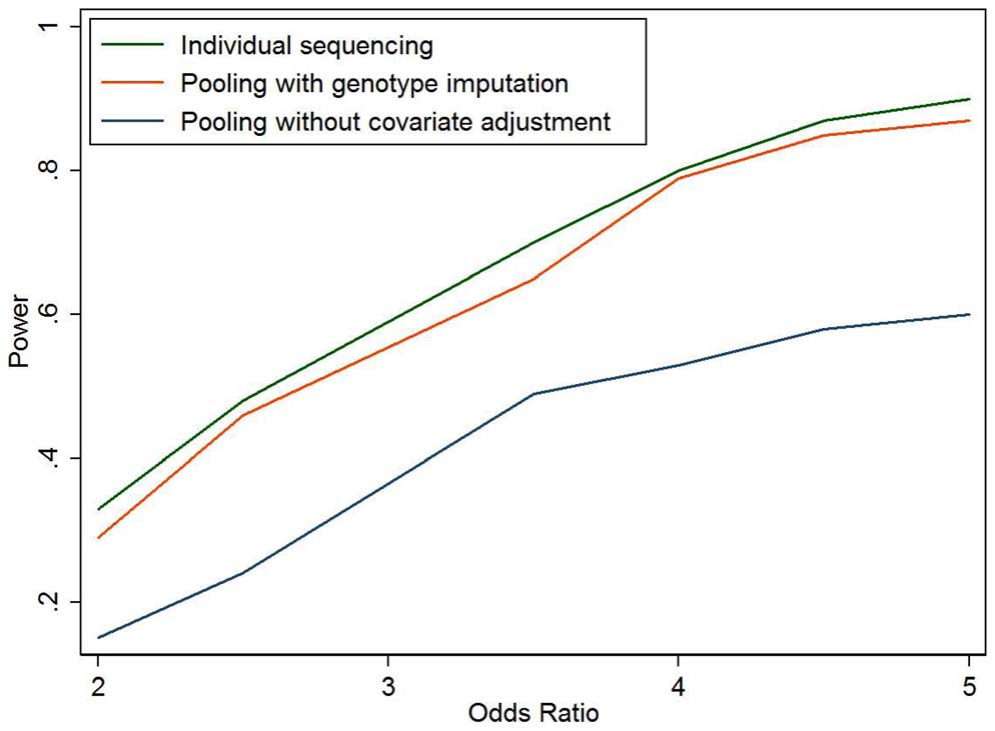
Power for individual sequencing of all samples, pooling with individual genotype imputed, and pooling without considering other risk factors. The simulation setting is described in Table 1, model 5, but with different odds ratio for the risk allele (OR_g_). Number of simulations is 200 for each setting.

**Figure 2:**
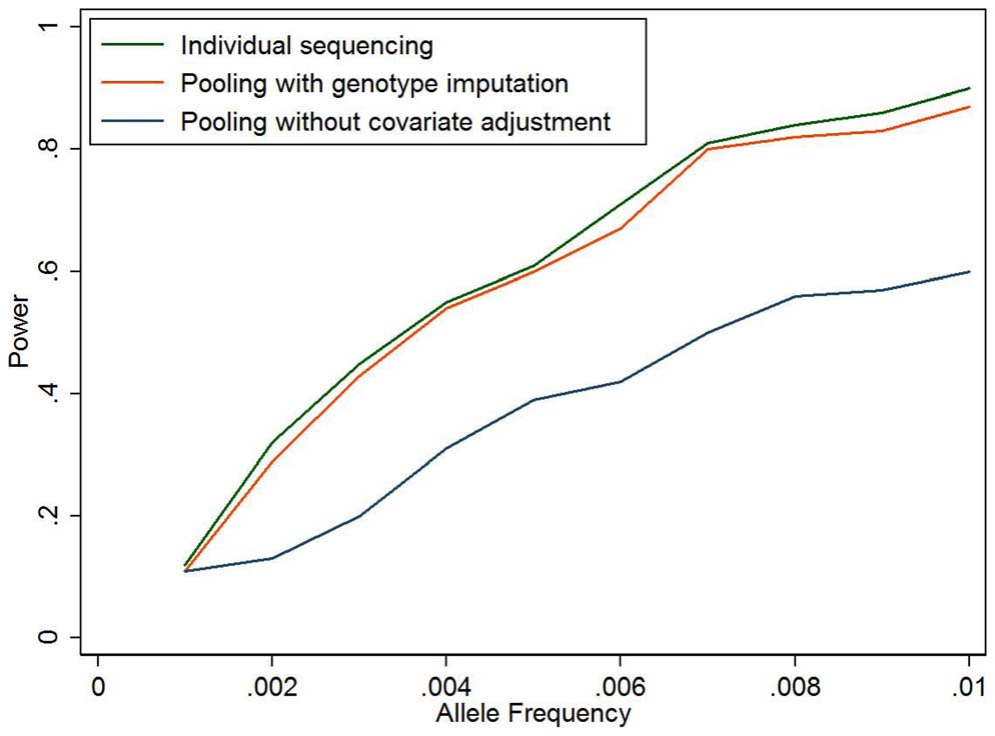
Power for individual sequencing of all samples, pooling with individual genotype imputed, and pooling without considering other risk factors. The simulation setting is similar to that described in Table 1, model 5, but with different risk allele frequency (RAF) with n = 5000 cases/controls, and OR_g_ = 2. Number of simulations is 200 for each setting.

**Figure 3:**
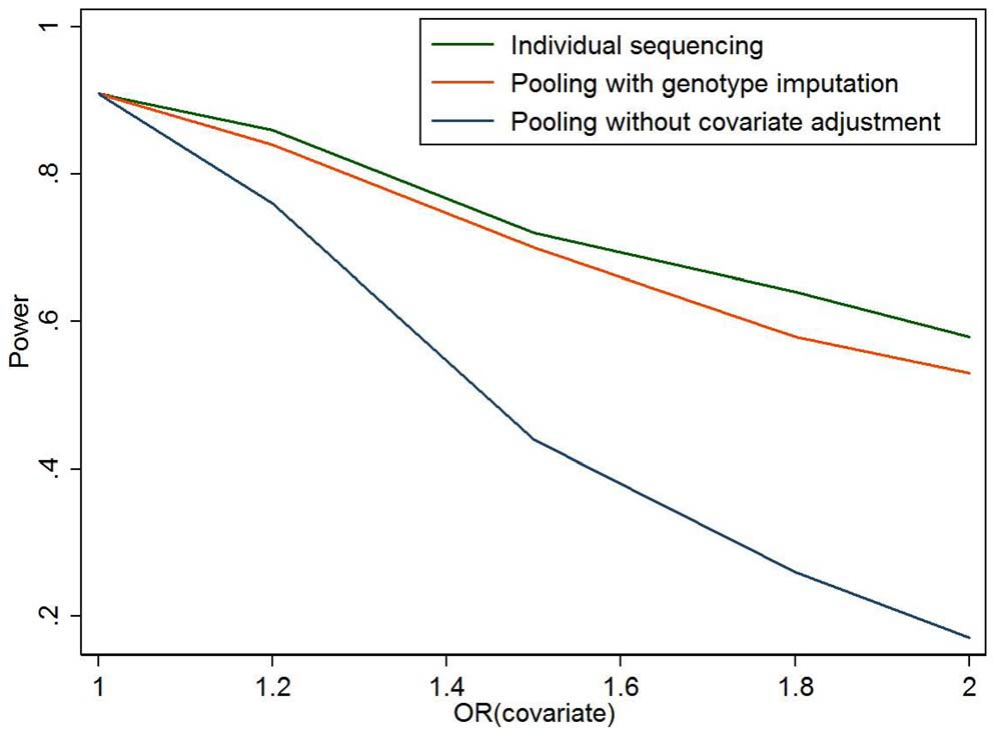
Power for individual sequencing of all samples, pooling with individual genotype imputed, and pooling without considering other risk factors. The simulation setting is described in Table 1, model 5, but with different odds ratio for the covariates (OR_z_). Number of simulations is 200 for each setting.

When the sequenced variant is causal (Table 3), our proposed method is slightly less powerful than individual sequencing of all samples, but can be much more powerful than standard pooling method ignoring covariate information. For example, when there is no correlation between genetic variant and other covariates (model 5), although the standard pooling method has appropriate type 1 error, it fails to take into account other risk factors contributing to the disease and therefore has lower power than multivariate models in our imputation-based method. Fig. 1 compares the power of the three approaches for different genetic effects. Our imputation-based method is about 2–5% lower in statistical power compared to individual sequencing, but has 15–30% more power than the standard pooling approach which cannot adjust for covariates. Fig. 2 compares the power at different causal allele frequencies with similar observation. We also assess the impact of effect size of covariates Z in the simulations (Fig. 3). When OR of Z is 1 (no effect), the three methods have almost the same power; when the OR increases, the power advantage of our imputation-based method over standard pooling becomes more and more significant.

**Table 3:** Power for multiple-imputation based pooling method (“pool_MI-prob_”), individual sequencing of all samples (“seq_all_”) and pooling without considering other risk factors (“pool_univariate_”).

Model	seq_all_	pool_univariate_	pool_MI_
5	.71	.42	.66
6	.63	.35*	.59
7	.42	.25*	.37
8	.52	.30*	.47
9	.71	.24*	.64
10	.50	.25*	.48

*. Power adjusted for the nominal false positive rates.

The significance level  = .05 (10^−4^ for model 11). Number of simulations is 1000.

We further evaluate the performance of our proposed method when only a subset of covariates is included in the analysis (Table 3, model 7), or when the effect sizes of the five risk factors vary (Table 3, model 8). We also apply a stringent threshold for significance (model 4 and 9), and our proposed method still maintains correct type 1 error rate and good statistical power, compared to other methods.

### Unequal proportion of samples in pool

When technical variability in sample preparation lead to unequal contribution of individual samples in the pool, estimated allele frequency of sequenced variant can be biased from pooled samples [8], [17]. Our proposed method still maintains reasonable type 1 error (model 3), while having higher power than standard pooling approach without covariate adjustment (.48 vs.23 and.25, respectively) (model 12).

### Impact of sequencing errors

We further evaluate the impact of sequencing errors that can potentially bias the disease-marker association. Our method incorporates the estimated sequencing error rate when imputing the individual genotypes from pooled DNA reads. The simulation results show that our method maintains correct type 1 error rate (Table 4).

**Table 4:** Type 1 error (model 2) and power (model 6) for multiple-imputation based pooling method with sequencing error rate of 0.5%, 1%, and 2%.

Model	Sequencing error rate		
	0.5%	1%	2%
2	.051	.053	.056
6	.66	.67	.62

Number of simulations is 1000.

## Discussion

DNA pooling is a cost effective alternative for genetic sequencing studies, but the standard pooling approach ignores covariate information that can lead to power loss or false positives. We have proposed a new study design for sample pooling that matches samples based on their covariates prior to sequencing and uses multiple imputation technique for association test. Simulation studies show that our approach can control the false positive rates in presence of confounders and improve power of association test compared to standard pooling approach or individual sequencing given the same cost.

Our new method tackles the problem of covariate adjustment by matching samples in the pooling stage. We consider a matching strategy based on quantiles of predicted probabilities. Other matching strategy can also be implemented, for example, K-means or K-median clustering [18] and nearest neighbor algorithm [19] using Euclidean distance (for continuous covariates) or Hamming distance (for discrete covariates). Such methods can separate potential outliers in the distribution of covariates, but often result in variable group sizes that may not be desired in practice. It has been shown that optimal data clustering with size constraint is computationally extensive, a “NP-complete problem” in computational complexity theory [20]. To obtain sample pools of equal size, we apply a greedy approach based on Euclidean distance. To create *l* pools, we first select *l* samples (centers) randomly, and assign rest of the samples one at a time to their closest pool defined by the minimum Euclidian distance to the center sample. If the closest pool reaches the desired pool size *K*, the sample will be assigned to the next closest pool. Simulations (data not shown) show slightly lower power and more variable estimates of genetic effect using this approach. In practice, well-matched samples can be pooled with equal pool size while “outlier” samples are sequenced individually. Another advantage of this unbalanced “blocked pooling” design is that the pools with a single individual can provide accurate estimate for sequence errors, and pools with a large number of individuals provide accurate estimate for allele frequency.

The advantage of using predicted probabilities over Euclidean distance is that it weighs the covariates by their estimated effect sizes in a model without the genetic variant. In presence of a confounding variable such as ethnicity, the estimated effect size of such variable can be biased in this model. However, given the current GWAS results for common variants and the low frequency of rare variants, we expect that a single variant can only explain a small proportion of total variance of outcome and the bias will be small. Our simulation results show that when the effect size of covariates vary, matching based on predicted probabilities performs slightly better than that based on Euclidean distance.

When imputing the genotypes for individuals in the pool, we assume HWE to derive the posterior distribution 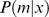. For analysis of low-frequency variants, the homozygote of minor alleles is often negligible and HWE has little impact on the validity of the imputation. Furthermore, the case-control sampling scheme can lead to violation of HWE when the variant is causal. In our pooling design, the cases and controls are pooled separately and the allele frequency is also estimated separately. Our simulation results, based on 1000 selected cases and controls, show correct type 1 error rate under different settings. However, when the disease model is not multiplicative, the HWE can be violated in cases which will lead to biased estimate for allele frequency and incorrect imputation, especially when the causal allele is not rare. In such case, the distribution of *P*(*m*) needs to be modified to reflect the true disease model.

Besides the assumption of HWE, our proposed method also relies on accurate estimation of allele frequency to draw valid imputations. In practice, unequal contribution of samples to the pool and sequencing errors can both lead to biased estimation. First, individual DNA samples may not be pooled with equal concentration due to technical variability [8], [17]. This bias can be minimized by careful quantification of DNA samples prior to pooling and choice of target enrichment method [5]. In our simulation study, we consider a scenario that the actual proportion of each sample can vary dramatically from (close to) 0 to 200% of the equal proportion of 1/N. Since the imputation is independent of disease outcome and the sampling error was randomly assigned to the pools, our proposed method still show false positive rate close to the nominal value. When the allele is causal, power from our method is not significantly impacted as well. Gautier et. al. [10] proposed a Bayesian hierarchical model to obtain accurate estimation of allele frequency with unequal proportion of samples in the pool. Second, sequencing errors are common in next-generation sequencing experiments. Besides the “blocked pooling” design we consider, Chen et al. [8] and Futschik and Schlotterer [7] proposed statistical methods to estimate the true allele counts from pooled samples with sequencing errors being taken into account. All these method can be incorporated into our proposed approach to improve genotype imputation.

There are also several practical issues for our proposed methods. First, we consider a pool size of 12 in our simulations. Gautier et. al. [10] showed that large pool size (e.g.,>40) can help to reduce the impact of unequal contribution of individuals to the pool. However, large pool size may also lead to worse matching on the covariates. We run simulations with different pool sizes (S1 Table), and find slightly increased type 1 error when the pool size is>50 at the presence of confounders. This result may vary by the actual distributions of covariates. When the confounder is discrete (e.g., ethnic groups), exact matching is possible and large pool size become more feasible. Second, literatures on multiple imputations (e.g., [16]) typically suggest a small number of imputations to use. We evaluate the impact of number of imputations (S2 Table). The results suggest that increasing the number of imputations does not change the performance of our method significantly. Third, the sequencing coverage needs to be considered given different pool size. In our simulations, we choose a pool size of 12 with ∼200X coverage. We expect that a larger pool size will require a greater coverage to achieve accurate estimate for allele frequencies. Fourth, in our simulations, we assumed all samples were processed in a single lane. For a study with large sample size but small pool size, multiple lanes may be needed. The sequencing error will then be estimated separately for each lane, but the allele frequency estimates can be averaged across lanes to achieve greater accuracy. The imputation procedure will be performed accordingly for pools in different lanes.

In our simulation study, we consider association test based on a single variant, which is often applied in targeted re-sequencing studies to identify or validate potential causal variants. Alternatively, analysis of low-frequency genetic variant (MAF <.01) often model multiple variants simultaneously within a gene or targeted region (e.g., [21]–[23]) to increase the power of association tests. Assuming such variants are independent, our method can be extended to these multi-locus tests, by imputing individual genotypes at each locus separately from the pooled sequence reads. A multi-marker test can then be applied to the imputed genotypes at multiple loci. A statistical method can also be developed to improve the accuracy of imputation if the linkage disequilibrium (LD) information in the region can be inferred from a reference panel such as the 1000 Genomes project.

In summary, we have proposed a new framework to match samples using the covariate information to create sequencing pools and use multiple imputation technique for association test that allows covariate adjustment. Our method is specifically designed to improve power to detect the disease-marker association using the cost effective DNA pooling approach, and correctly control the false positives in presence of confounding effect. A sample Stata code is available freely at http://www.biostat.umn.edu/~wguan/software/, which demonstrates the pool creation and multiple imputation pipeline. We expect our method will aid analyses of sequencing-based association studies for complex traits.

## Appendix

**Figure 4:**
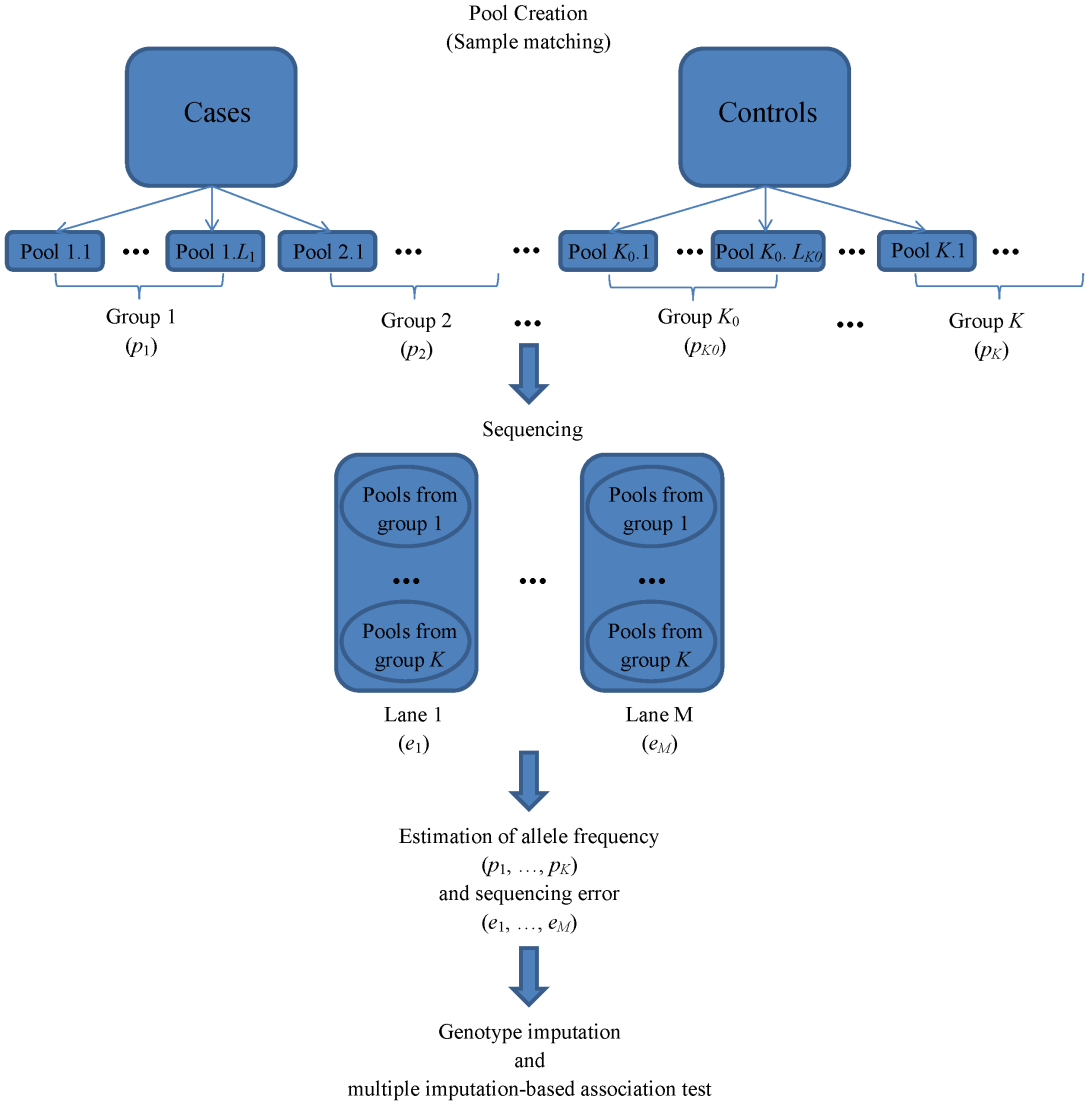
Design of DNA pooling with sample matching. After sample matching and pool creation, the pools are grouped into *K* groups, with allele frequency in each group denoted by (*p*_1_, …, *p_K_*). Pools from the same groups are randomly distributed into *M* lanes, with sequencing errors (*e*_1_, …, *e_M_*).

The proposed design for DNA pooling is described in Fig. 4. The samples will be matched based on their covariate values to create DNA pools. Pools with similar covariate values and the same case/control status will be further grouped together. We assume the allele frequency of risk allele is constant for samples within each group of pools. When multiple lanes are needed, the pools from the same group will be randomly distributed across lanes to minimize potential batch effects. We assume the sequencing error rate is constant for pools within the same lane.

We first consider a DNA pool with *K* individuals. Let *p* and *q* = 1 - *p* be the frequencies of alleles *a* and *A*, respectively. Let *m* be the number of allele *a* in the pool of 2*K* alleles. Assuming Hardy-Weinberg equilibrium (HWE), *m* follows a binomial distribution Bin(2*K*, *p*). Let 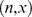 be the observed sequence data for the pool, where *n* is the total number of reads at the locus and *x* the number of reads with allele *a*. Denote *e* the sequencing error rate, and then 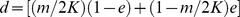 the probability of observing a read of allele *a*. The joint probability:
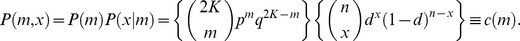
and the posterior distribution of *m* given observed reads *x* is 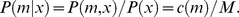
where 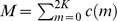. Note that *c*(0) = *c*(2*K*) = 0.

To estimate the allele frequency *p* and sequence error rate *e*, an EM algorithm was described by Wang et al. [9]. We extend the algorithm to allow joint estimate of *p*s and *e*s from multiple groups of pools (each group with the same value of covariates and case/control status) and multiple lanes. Let *i* denote the lane, and assuming the sequence error is consistent for pools within the same lane, we denote the error rate by 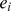. We denote the allele frequency in group *k* by 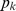. In the simple case where no covariate is considered, *k* = 1 or 2 for group of cases and controls, respectively. For pool *j*, let 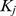 denote the pool size. To reduce potential batch effects, the pools in the same group will be distributed randomly across all the lanes, i.e., pool *j* is nested within group *k* and within lane *i*, but *k* is in general not nested within *i*. Let L*_k_* denote the number of pools in group *k*, and L*_i_* denote the number of pools in lane *i*.

1. E step:, for the *j*-th pool in group *k*, lane *i*.

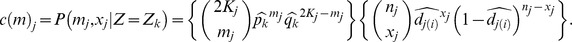
where, 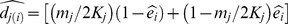
and, 
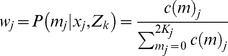

2. M step:

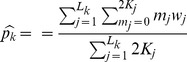


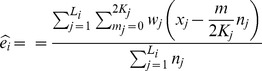

When the covariates are continuous, no exact match can be done for pooling samples. In practice, we can divide the samples by quantiles of matching score, e.g., 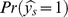 as described in “Methods” to achieve large sample size in each group of pools to estimate the allele frequency 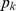. In our simulations, we divide the case pools and control pools into 4 groups each, i.e., L*_k_*≈20, to estimate the allele frequency. This approach will oversimplify the distribution of allele frequencies, but performs fairly well in our simulation results.

## Supporting Information

S1 Table Type 1 error (model 2) for pool size of 12, 30, and 50. Number of simulations is 1000.
(DOCX)

S2 Table Type 1 error (model 2) and power (model 6) for multiple-imputation based pooling method using 5, 10, and 100 imputations. Number of simulations is 1000.
(DOCX)
