# Supplementary material for: Design of DNA Pooling to Allow Incorporation of Covariates in Rare Variants Analysis
Source: PLoS One. 2014 Dec 8;9(12):e114523. doi: 10.1371/journal.pone.0114523 (PMC4259344; doi:10.1371/journal.pone.0114523)
Supplement: S1 Table — Type 1 error (model 2) for pool size of 12, 30, and 50. Number of simulations is 1000. (DOCX) [file pone.0114523.s001.docx]

Supplemental table S1. Type 1 error (model 2) for pool size of 12, 30, and 50. Number of simulations is 1000.

| Model | Pool size | | |
| --- | --- | --- | --- |
|  | 12 | 30 | 50 |
| 2 | .052 | .055 | .065 |
