# Supplementary material for: Design of DNA Pooling to Allow Incorporation of Covariates in Rare Variants Analysis
Source: PLoS One. 2014 Dec 8;9(12):e114523. doi: 10.1371/journal.pone.0114523 (PMC4259344; doi:10.1371/journal.pone.0114523)
Supplement: S2 Table — Type 1 error (model 2) and power (model 6) for multiple-imputation based pooling method using 5, 10, and 100 imputations. Number of simulations is 1000. (DOCX) [file pone.0114523.s002.docx]

Supplemental table S2. Type 1 error (model 2) and power (model 6) for multiple-imputation based pooling method using 5, 10, and 100 imputations. Number of simulations is 1000.

| Model | Number of imputations | | |
| --- | --- | --- | --- |
|  | 5 | 10 | 100 |
| 2 | .052 | .047 | .057 |
| 6 | .65 | .64 | .63 |
